# Structural Evolution and Mechanical Modulation of C_f_/SiC Interfaces During PIP Ceramization: A ReaxFF Molecular Dynamics Study

**DOI:** 10.3390/polym18060702

**Published:** 2026-03-13

**Authors:** Yue Zhan, Xudong Wang, Kang Guan, Ming Lv, Cheng Peng, Xiaohui Yang, Longteng Bai

**Affiliations:** 1 School of Materials Science and Engineering, South China University of Technology, Guangzhou 510640, China; tenesseewhut@gmail.com (Y.Z.); immlv@scut.edu.cn (M.L.); mscpeng@scut.edu.cn (C.P.); 2 Xi’an Aerospace Propulsion Institute, Xi’an 710100, China; wxdfine@163.com (X.W.); yangxiaohui925@163.com (X.Y.); bailt9520@163.com (L.B.)

**Keywords:** pyrolysis kinetics, C_f_/SiC composites, ReaxFF molecular dynamics, interfacial evolution, mechanical failure mechanism

## Abstract

The precursor infiltration and pyrolysis (PIP) route is widely adopted to fabricate carbon fiber-reinforced silicon carbide (C_f_/SiC) composites; however, the atomic-scale restructuring of the pyrolytic carbon/silicon carbide (PyC/SiC) interface during ceramization—and its impact on mechanical integrity—remains elusive. Here, reactive molecular dynamics (ReaxFF MD) simulations elucidate the coupled thermochemical–mechanical evolution of polycarbosilane (PCS) precursors on PyC substrates with orientation angles (OAs) of 0°, 25°, 55°, and 85°. Dynamic pyrolysis triggers a pivotal transition from sp^2^ to sp^3^ hybridization at the interface. High-OA substrates (55° and 85°) present a dense population of reactive edge sites, fostering extensive cross-interfacial covalent bonding. Subsequent shear loading reveals that these pyrolysis-induced chemical bridges govern failure modes, shifting from interlayer sliding dominated by weak non-bonded interactions (0°) to ductile fracture featuring uniform plasticity and crack deflection. The OA = 55° interface attains a theoretical peak shear strength of 15 GPa and exhibits the most favorable combination of high strength and ductile failure under tensile loading, owing to an optimal balance between reactive site availability and interlayer steric openness. In contrast, the OA = 85° interface, despite comparable peak stress, fails via brittle crack penetration into the SiC matrix. By correlating atomistic structure with macroscopic performance, this study provides a bottom-up framework for engineering C_f_/SiC composites via interfacial texturing and optimized pyrolysis protocols.

## 1. Introduction

In carbon fiber-reinforced silicon carbide (C_f_/SiC) composites fabricated by the precursor infiltration and pyrolysis (PIP) route [1,2,3,4,5,6], a pyrolytic carbon (PyC) interphase is deliberately deposited on the fiber surface to serve two mechanical functions: (i) deflecting matrix cracks along the interface and (ii) accommodating the coefficient-of-thermal-expansion (CTE) mismatch between fiber and matrix [7,8]. During ceramization, however, the precursor-to-ceramic conversion entails substantial volumetric shrinkage, thereby generating internal stresses that concentrate at the PyC interphase [9]. Because the interfacial microstructure is established during this transient thermochemical stage, the ultimate load-transfer capability of the composite is fundamentally encoded in the atomic-scale events occurring at the precursor/PyC boundary. Precisely how this boundary restructures during pyrolysis—and how such restructuring dictates mechanical integrity—remains unresolved.

The interface is therefore a critical determinant of composite performance [10,11,12,13,14]. Numerous studies have modified PyC characteristics to enhance the macroscopic properties of C_f_/SiC laminates. Experimental observations show that high-temperature treatments can markedly alter the interfacial bonding state, producing matrix cracking, interfacial debonding, and a structurally interlocked PyC–SiC interface that collectively improve tensile and flexural toughness [15,16]. Moreover, the intrinsic texture of the PyC layer—from low-textured (LT) to high-textured (HT)—strongly influences bulk mechanical response [17]. Yet static post-mortem characterisation cannot capture the transient atomic rearrangements driven by ceramization, leaving the mechanistic origin of these correlations obscure.

Molecular dynamics (MD) has emerged as a powerful tool for interrogating polymer pyrolysis at atomic resolution [18,19,20,21,22]. Naserifar et al. reproduced the temperature–dependent ceramization of hydridopolycarbosilane (HPCS) precursors, achieving excellent agreement with experiment [23,24]. Gao et al. simulated mixed HPCS/PMHS feeds and obtained amorphous SiOC structures consistent with spectroscopy [25], while Lu et al. elucidated side-group and temperature effects on SiOC formation [26]. Recently, the application of ReaxFF MD has been further expanded to investigate the complex free carbon evolution and high-temperature pyrolysis behavior during the conversion of polymer precursors into ceramics [27,28]. Despite this progress, most simulations neglect interactions between the precursor and a solid interface. Existing MD studies of composite interfaces concentrate on mechanical evaluation alone: Zhan et al. compared graphene adhesion on Si- versus C-terminated SiC [29]; Zhou et al. related PyC crystallite size to C/PyC shear failure [30]; and Niu et al. examined how carbon-layer density and spacing influence SiCf/SiC shear strength [31]. Most relevant, Wang et al. demonstrated that the orientation angle (OA) of graphene layers within PyC governs interfacial failure, ranging from localized sliding to uniform plasticity [32]. Furthermore, MD-based simulations have been employed to investigate the interfacial mechanical behavior and toughening mechanisms of PyC interphases [33,34,35]. Crucially, all of these models adopt a predefined, static interfacial topology that implicitly represents the post-pyrolysis state.

This decoupling of interface formation from mechanical assessment obscures the causal link between pyrolysis conditions (e.g., temperature, precursor chemistry) and eventual load transfer. Optimising PIP processing therefore demands a simulation framework that captures both the reactive chemistry of precursor decomposition and the subsequent mechanical response within a single atomistic workflow.

Here we develop such a coupled thermochemical–mechanical protocol using ReaxFF MD. To accurately represent the actual PIP manufacturing environment, polycarbosilane (PCS) and pyrolytic carbon (PyC) are utilized to construct the interface model, corresponding to the predominant preceramic polymer and the standard interphase in typical C_f_/SiC composites, respectively. This reactive potential is specifically chosen to overcome the limitations of traditional empirical force fields in simulating the complex bond dissociation and cross-linking reactions during high-temperature ceramization. Polycarbosilane (PCS)/PyC interface models with four representative orientation angles (OA = 0°, 25°, 55°, 85°) are first subjected to reactive pyrolysis at 2000 K, during which interfacial bond evolution, sp^2^ →sp^3^ hybridisation transitions, and cross-interface covalent bridge formation are monitored in real time. The resulting structures are converted to amorphous-SiC/PyC interfaces, and their tensile and shear responses are subsequently evaluated. We show that

(i) Pyrolysis induces a pronounced sp^2^-to-sp^3^ transition whose extent scales with the OA-dependent density of reactive edge sites;

(ii) The OA = 55° configuration develops the densest cross-interfacial covalent network through an optimal balance of site reactivity and steric accessibility; 

(iii) These pyrolysis-formed chemical bridges transform the failure mode from weak interlayer sliding to ductile fracture with crack deflection.

By establishing an atomistically resolved linkage between PIP processing conditions and intrinsic interfacial mechanics, this study provides a bottom-up framework for engineering C_f_/SiC composites via interfacial texturing and pyrolysis optimization.

## 2. Simulation Methods

The ReaxFF potential describes interatomic interactions through bond order (BO). The total potential energy of the system is the sum of various energy terms, as shown in Equation (1):

(1) $$E_{\mathrm{system}}\;=\;E_{\mathrm{bond}}\;+\;E_{\mathrm{over}}\;+\;E_{\mathrm{under}}\;+\;E_{\mathrm{val}}\;+\;E_{\mathrm{pen}}\;+\;E_{\mathrm{tors}}\;+\;E_{\mathrm{conj}}\;+\;E_{\mathrm{vdWaals}}\;+\;E_{\mathrm{Coulomb}}$$

where *E*_system_ and *E*_bond_ represent the system energy and bond energy, *E*_over_, *E*_under_, *E*_val_, *E*_pen_, *E*_tors_, *E*_conj_, *E*_vdWaals_, and *E*_Coulomb_ represent the over-coordination energy, under-coordination energy, valence angle energy, penalty energy, torsion energy, conjugation energy, van der Waals Energy and coulomb energy [36]. The specific ReaxFF parameter set used in this study was developed by Newsome et al. [37] for the Si/C/O/H system. This parameterization was trained against density functional theory (DFT) calculations of bond dissociation curves, angle distortion energies, and equation-of-state data for SiC polymorphs, as well as experimental heats of formation. It has been successfully applied to simulate the oxidation of silicon carbide [37] and the pyrolysis of polycarbosilane-type precursors [25], demonstrating good transferability to the Si–C–H chemistry relevant to the present PIP process.

**Figure 1:**
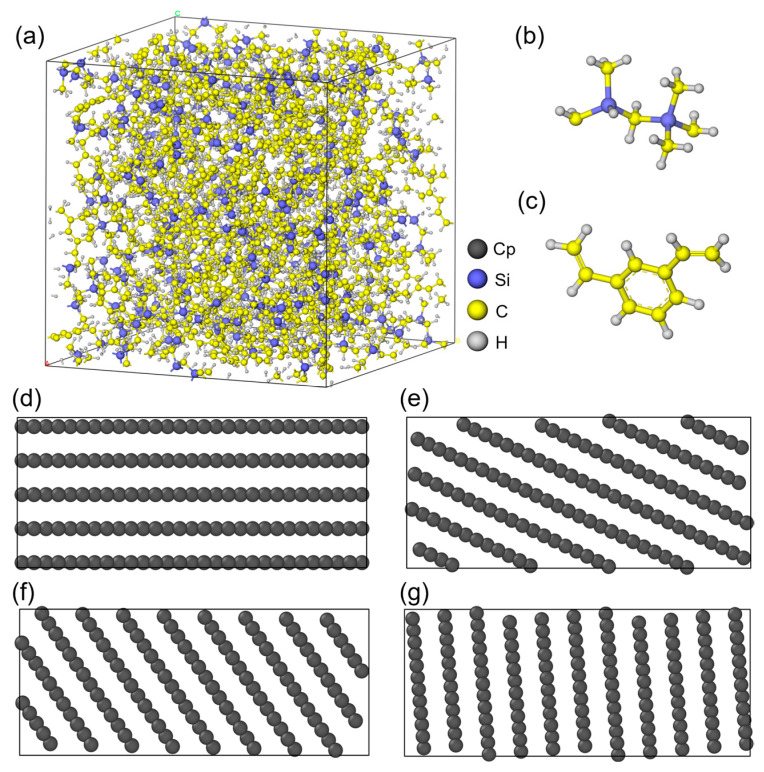
Atomic models used in this study: (**a**) Cross-linked PCS model with a cross-linking degree of 91.25%; (**b**) PCS repeating unit; (**c**) DVB cross-linker molecule; (**d**–**g**) PyC layers with different orientation angles (OA): (**d**) OA = 0°, (**e**) OA = 25°, (**f**) OA = 55°, (**g**) OA = 85°. Blue, yellow, and grey spheres represent Si, C, and H atoms.

**Figure 2:**
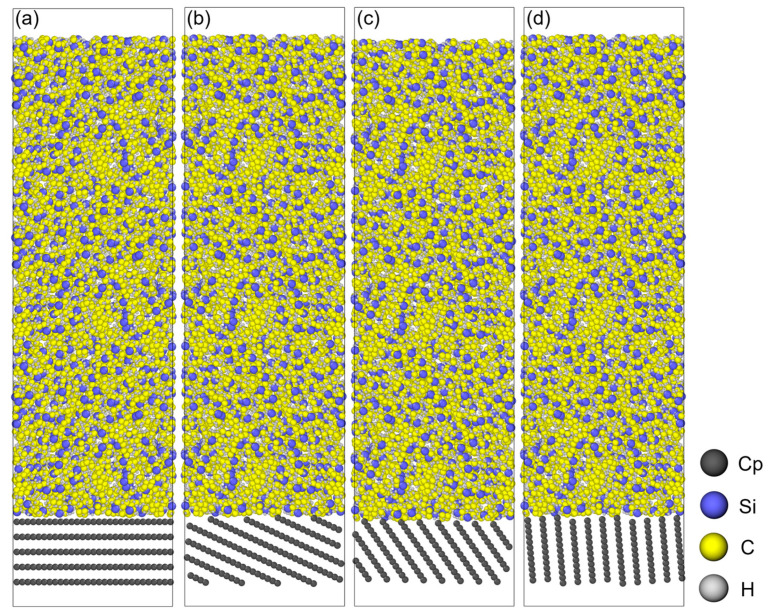
PCS/PyC interface models with different orientation angles: (**a**) 0°; (**b**) 25°; (**c**) 55°; (**d**) 85°.

First, the PCS molecular chain is built using Materials Studio 2020 based on polycarbosilane repeating units (Figure 1b). Then, the cross-linking simulation with divinylbenzene (DVB) is performed (Figure 1c). The obtained polymer chain has a cross-linking degree of 91.25% and a total of 4830 atoms. Subsequently, the Amorphous Cell module is used to construct the periodic polymer box at 298 K. After energy minimization via the Forcite module, the initial model is obtained with a side length of 35.62 Å and a density of 1.108 g/cm^3^ (Figure 1a). To meet the size requirements for subsequent mechanical simulations, this model is expanded along the *z*-axis to 35.62 × 35.62 × 106.86 Å. Meanwhile, graphite structures with a size of 35.62 × 35.62 × 15 Å are built as PyC models. These models have an interlayer spacing of 3.35 Å and orientation angles of 0°, 25°, 55°, and 85° (Figure 1d–g). Finally, the PCS/PyC interface models with different orientation angles are established (Figure 2).

To emulate the precursor architecture, the initial PCS/PyC interface systems were subjected to energy minimization via the conjugate gradient (CG) algorithm. The CG method is used to iteratively adjust atomic coordinates to minimize the potential energy of the system and eliminate unreasonable local interatomic contacts. In this study, the energy tolerance, force tolerance, and maximum number of iterations were set to 1.0 × 10^−4^, 1.0 × 10^−6^ eV/Å, and 10,000. Subsequent structural relaxation was achieved through a 50 ps NPT ensemble simulation at 300 K. To initiate the pyrolysis process, the equilibrated systems were linearly ramped to 2000 K at a heating rate of 0.1 K/fs within an NVT ensemble. This was followed by an 83 ps isothermal hold, yielding a cumulative pyrolysis duration of 100 ps. The temperature of 2000 K was selected to accelerate the reaction kinetics within the limited MD timescale, which is a common practice in ReaxFF simulations to observe polymer-to-ceramic conversion [24]. As discussed in Section 3.5, this temperature ensures sufficient interfacial cross-linking while avoiding excessive fragmentation, providing a representative view of the thermochemical evolution. For the 25° model, additional NVT simulations are conducted at 1500 K and 2500 K. To simulate the PIP process, volatile byproducts (e.g., H_2_, CH_4_, and other low-molecular-weight gaseous species) were periodically removed from the simulation box. Specifically, a custom script was employed to identify and delete free gas molecules based on their bonding topologies after each 50 ps NVT relaxation phase, accurately mimicking the outgassing effect in a real PIP furnace. Concurrently, to simulate the densification of SiC on the PyC surface, a reflective wall was applied at the upper z-boundary, moving along the negative *z*-axis at a controlled velocity of 0.0001 Å/fs. This step was repeated until no small molecule gases remained. Subsequently, the reflective wall was removed, and the system was cooled to 300 K at a rate of 0.1 K/fs. To eliminate artificial residual stresses introduced by the uniaxial compression, the system was fully relaxed in an NPT ensemble (300 K, 1 atm) for 50 ps. Importantly, the final density of the generated amorphous SiC (a-SiC) phase was verified to be consistent with typical polymer-derived ceramics (e.g., 2.6~2.7 g/cm^3^), thus validating the physical rationality of the densification step.

The thermochemical evolution of the PCS/PyC interface was captured using the ReaxFF reactive force field, which permits bond breaking and formation during pyrolysis. For the subsequent mechanical evaluation, however, the force field was switched to traditional empirical potentials—Tersoff for the SiC region and cross-interfacial covalent bonds (C–C and Si–C), and AIREBO (with a cutoff radius of 1.92 Å) for the PyC region. No direct parameter conversion was performed between ReaxFF and AIREBO/Tersoff. Instead, the final structure obtained from ReaxFF pyrolysis was transferred to the mechanical model, and atom pairs with BO > 0.3 were retained as stable covalent bonds. This transition was necessitated by the known tendency of ReaxFF to produce unphysical bond rupture events under large strains far from the equilibrium configurations for which it was parameterized [36].

To ensure physical fidelity during mechanical loading, a rigorous force field mapping procedure was implemented. First, the dynamic bonding topology at the end of the ReaxFF pyrolysis simulation was frozen to evaluate the intrinsic mechanical strength of the specific cross-linked network formed. Atoms connected by a bond order (BO) exceeding 0.3—a statistically established threshold for stable covalent bond formation in carbon-silicon systems—were assigned covalent interactions in the Tersoff potential. Second, the hybridization state of each interfacial carbon atom was determined from its coordination number (CN): CN = 2 (sp^1^), CN = 3 (sp^2^), and CN = 4 (sp^3^). These assignments were preserved during the transition. Third, the mapped structure was subjected to a multi-stage energy minimization (conjugate gradient followed by the FIRE algorithm) until the maximum per-atom force fell below 10^−4^ eV/Å. We acknowledge two limitations of this approach: (i) dynamic charge redistribution during mechanical loading is neglected, and (ii) the Tersoff potential may overestimate the stiffness of amorphous SiC relative to more accurate ab initio benchmarks. Consequently, the absolute stress values reported herein should be interpreted as theoretical upper bounds for ideal, defect-free interfaces, rather than as direct predictions of experimental interfacial strengths.

For mechanical simulations, the AIREBO potential with a cutoff radius of 1.92 Å is used for the PyC layer. The Tersoff potential is used to describe the interactions within SiC and at the SiC/PyC interface. These potentials are adopted to accurately reflect the effect of interfacial chemical reactions on mechanical properties [32,38]. First, the interface models are relaxed at 300 K in an NVT ensemble for 50 ps. Then, another 50 ps relaxation is performed in an NPT ensemble. For mechanical simulations, a 4 Å layer at the top of the SiC/PyC model is set as the moving layer, and a 4 Å layer at the bottom is fixed. The loading direction for tensile tests is along the positive *z*-axis. According to the PyC orientation angle, the loading direction for shear tests is set as the negative *x*-axis. The loading velocity is 0.01 Å/ps. Periodic boundary conditions are applied in the x and y directions. The time step for all mechanical simulations is 0.0005 ps.

It is necessary to detail the main constraints and limitations involved in our simulation approach. First, the heating rate of 0.1 K/fs (10^14^ K/s) is approximately 10 orders of magnitude faster than typical furnace ramp rates (~1–10 K/min). Such accelerated kinetics are inherent to atomistic simulations and may shift reaction onset temperatures or alter the branching ratios among competing decomposition pathways. Nevertheless, previous ReaxFF studies have demonstrated that the dominant bond-breaking sequences and final product compositions are qualitatively preserved at these rates [23,24], supporting the use of our results for mechanistic insight if not for quantitative kinetic parameters. Second, the simulation cell dimensions (35.62 × 35.62 × ~120 Å) and the PyC substrate thickness (~15 Å) are necessarily limited by computational cost. Finite-size effects may constrain the diffusion pathways of gaseous byproducts and suppress long-wavelength stress fluctuations during mechanical loading. Consequently, the absolute values of peak stress and failure strain should be interpreted with caution, while the relative trends across different OAs configurations are expected to be robust. Third, the mechanical loading rate of 0.01 Å/ps corresponds to a strain rate on the order of 10^8^–10^9^ s^−1^, which is 6–8 orders of magnitude higher than quasi-static experimental conditions. At such rates, thermal activation of defect nucleation and crack propagation is suppressed, leading to an overestimation of peak stress. Despite these quantitative limitations, the comparative framework established here—where all models are subjected to identical simulation conditions—enables reliable identification of the OA-dependent structure–property relationships.

## 3. Results and Discussion

### 3.1. Bond Number Evolution During Precursor Pyrolysis

**Figure 3:**
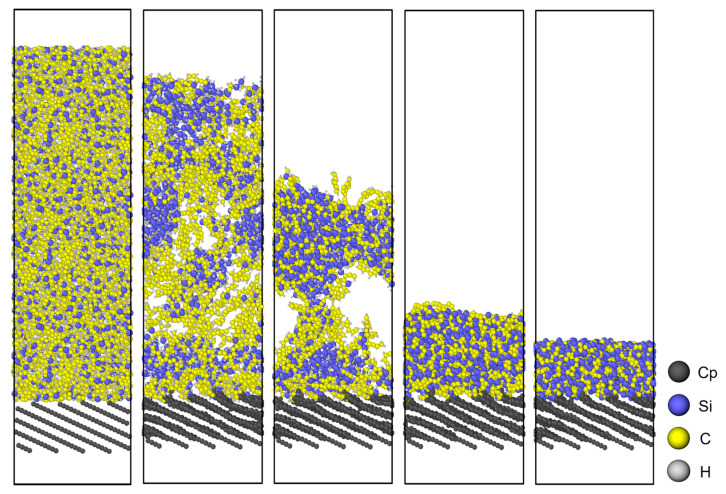
Schematic diagram of the pyrolysis simulation for the PCS/PyC interface model. Black spheres (C_p_) represent carbon atoms in the PyC layer.

**Figure 4:**
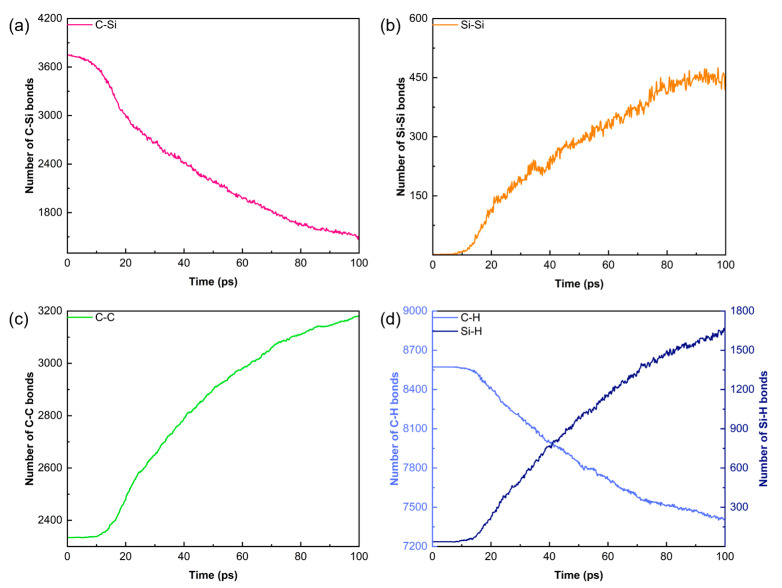
Time evolution of key bond populations during the 100 ps pyrolysis process: (**a**) C–Si; (**b**) Si–Si; (**c**) C–C; (**d**) C–H and Si–H.

Figure 3 illustrates the atomic configuration of the PCS/PyC interface during pyrolysis, while Figure 4 quantifies changes in the principal bond populations (C–Si, Si–Si, C–C, C–H, and Si–H) over the 100 ps trajectory. Because the simulation is performed in an NVT ensemble, both the atom count and the cell volume remain constant, allowing direct comparison of bond statistics throughout the run.

The initial decrease in C–Si bonds signals cleavage of the PCS backbone (–Si–CH_2_–Si–). This rupture proceeds slowly during the early ramp, but accelerates once the temperature reaches 2000 K, where rapid depolymerisation produces a surge of Si and C radicals. These highly reactive fragments subsequently drive extensive atomic rearrangement and cross-linking.

Concomitant scission of C–Si and C–H bonds liberates carbon-centred radicals that recombine to generate a steep rise in C–C connectivity, indicating progressive graphitisation. Owing to the excess of carbon relative to silicon, a fraction of the under-coordinated Si atoms dimerise to form a modest number of Si–Si bonds.

Because volatile species are not removed during this 100 ps NVT segment, mobile H atoms are transiently trapped by unsaturated Si centres, leading to an apparent increase in Si–H bonds. In an actual PIP furnace these Si–H-containing fragments would either desorb (e.g., as SiH_4_, Si_2_H_6_) or decompose further once gaseous products are purged. The subsequent gas removal cycles implemented later in our workflow faithfully replicate this experimentally observed outgassing.

### 3.2. Interfacial Chemistry and Hybridisation Evolution During Pyrolysis

**Figure 5:**
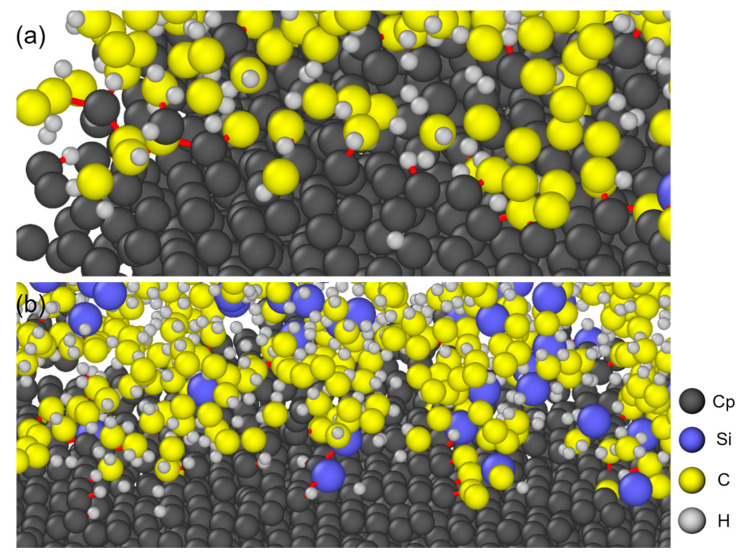
Bonding states of C–H, Cp–C, Cp–H, and Cp–Si at the interface for OA = 25°: (**a**) C–H and Cp–H and Cp–C; (**b**) C–H, Cp–H, Cp–Si and Cp–C The bond identification is based on the bond order (BO) criteria within the ReaxFF potential. The red lines represent chemical bonds.

Figure 5 visualises the bonding network that develops at the PCS/PyC interface with an orientation angle (OA) of 25° after the 100 ps pyrolysis run. Edge carbon atoms on the PyC side (labelled Cp) are clearly the most reactive sites. Within the ReaxFF framework, this enhanced reactivity arises from their lower coordination numbers, which result in higher under-coordination penalties and greater available bond order for forming new covalent bonds relative to the fully saturated basal-plane carbons.

**Figure 6:**
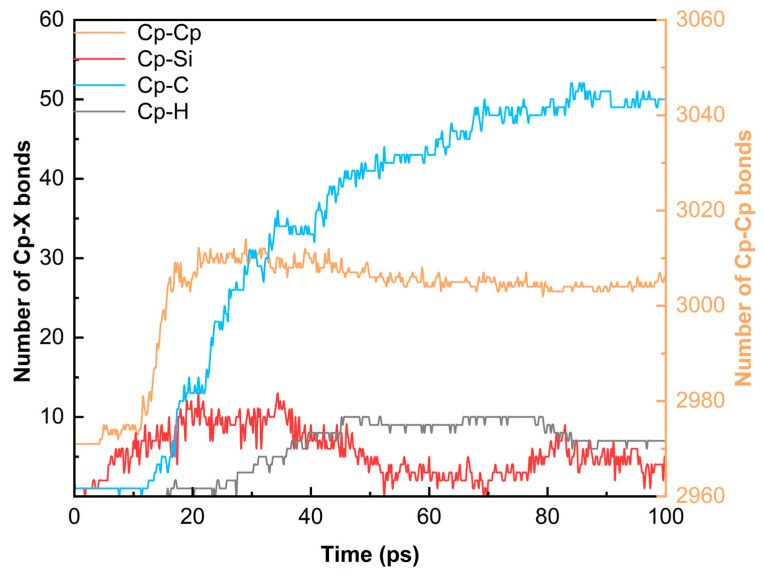
Time-dependent evolution of interfacial chemical bonds (Cp–X) for the OA = 25° interface during the 100 ps pyrolysis process. (Cp represents carbon atoms in the pyrolytic carbon layer; X = Cp, Si, C, and H atoms).

Figure 6 reveals the quantitative changes in interfacial bonds for the 25° model over time. As many active radicals (such as –CHx and –SiHx) form, the interfacial bonding enters a rapid growth stage. During the isothermal stage, the number of Cp–C bonds increases steadily. In contrast, the number of Cp–Si bonds peaks at around 30 ps and then exhibits an unsteady decrease. This transient spike in Cp–Si bonds represents a kinetically driven intermediate state, which is subsequently displaced by thermodynamically more stable Cp–C bonds (bond dissociation energy ~346–376 kJ/mol vs. ~318 kJ/mol for Si–C) during the structural reorganization of the carbon-rich precursor. The newly formed Cp–Si bonds are thermodynamically outcompeted, prompting Si atoms to either form clusters or detach from the interface. This represents the continuous breaking and recombination of bonds during pyrolysis. In addition, the number of Cp–Cp bonds increases throughout the process. It can be inferred that high-temperature pyrolysis promotes the structural reorganization of PyC to reduce surface energy, driven by point defects and edge carbon atoms.

**Figure 7:**
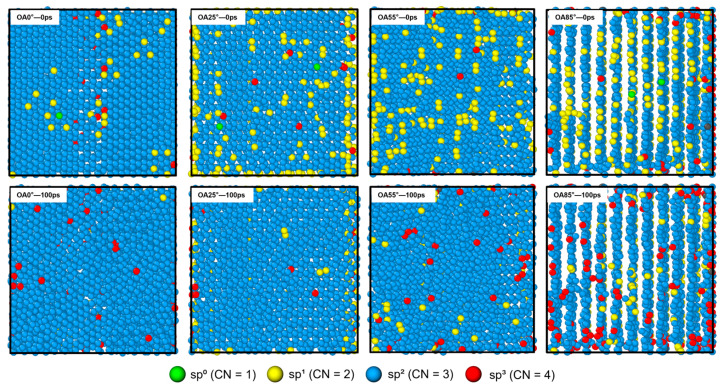
Top views of the hybridization distribution of Cp atoms at interfaces with different OAs during the 100 ps pyrolysis process.

Figure 7 shows the top views of PCS/PyC interfaces with different OAs before and after 100 ps of pyrolysis. To show the structural evolution clearly, Cp atoms are colored based on their sp hybridization states. At the start, Cp atoms at the OA = 0° interface are mainly sp^2^ hybridized, forming a nearly complete hexagonal network. Atoms with sp^0^ and sp^1^ hybridization are mostly found at vacancy edges and surface defects. For OA = 25°, 55°, and 85°, the number of sp^1^ atoms increases with OA and they align along the interlayer edges. These atoms represent highly reactive sites. After 100 ps of pyrolysis, the hybridization states of Cp atoms on all surfaces change significantly. In particular, low-coordinate atoms (sp^0^, sp^1^) largely disappear. These atoms either bond with active small molecules or increase their coordination through surface reconstruction. The change from sp^1^ and sp^2^ to sp^3^ hybridization represents a reaction mechanism of the pyrolytic carbon: edge atoms form stable sp^3^ structures with PCS products. This sp^3^ cross-linked network creates a strong covalent bonding bridge. In addition, the PyC substrate (blue atoms) maintains its structure despite the intense reactions at 2000 K.

**Figure 8:**
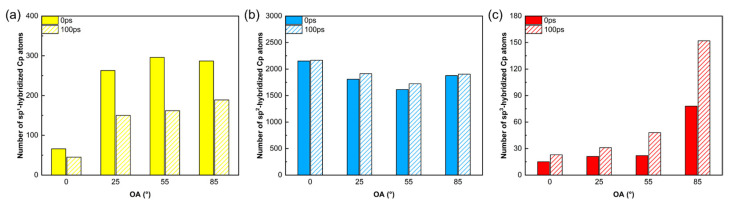
Number of sp-hybridized interfacial Cp atoms for different OAs during the 100 ps pyrolysis process: (**a**) sp^1^; (**b**) sp^2^; (**c**) sp^3^. sp^0^-hybridized atoms are excluded due to their negligible quantity (≤3 atoms) and complete disappearance after 100 ps.

Figure 8 quantifies the evolution of different sp-hybridized Cp atoms. These statistics corroborate the structural findings in Figure 7. First, the number of highly active sp^1^ hybridized atoms decreases significantly post-pyrolysis, particularly in high-angle systems (OA = 25°, 55°, and 85°), confirming that PyC interface edge defects actively participate in bonding reactions. Second, the number of sp^3^ hybridized atoms, which serve as covalent bridges, increases significantly, with the effect becoming more pronounced as the OA increases. In the OA = 85° system, the sp^3^ count nearly doubles, indicating that abundant edge sites in high-angle systems effectively promote a cross-linked network. Finally, the total number of sp^2^ hybridized atoms remains stable (1500–2000), proving that the graphene network within the PyC substrate stays intact, ensuring the structural integrity required for effective load transfer.

**Figure 9:**
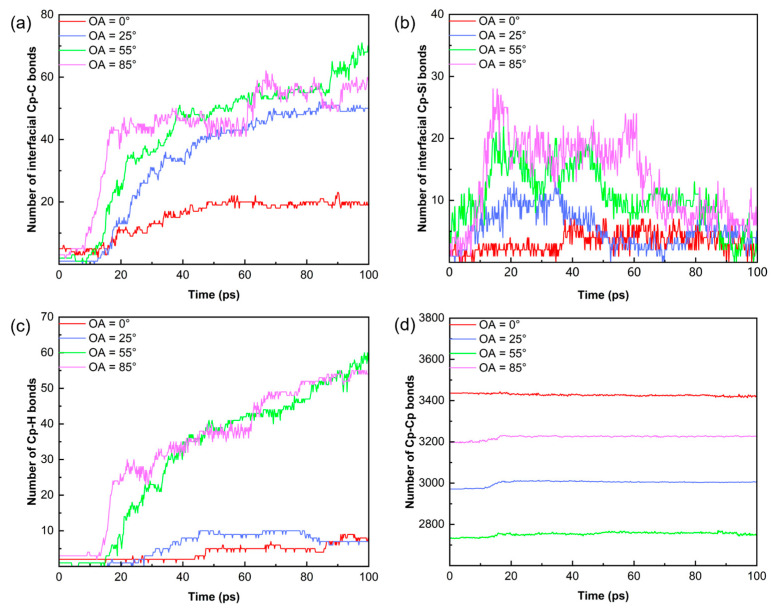
Evolution of Cp–X bond numbers for interfaces with different OAs during 100 ps of pyrolysis: (**a**) Cp–C bond; (**b**) Cp–Si bond; (**c**) Cp–H bond; (**d**) Cp–Cp bond.

Figure 9 illustrates the evolution of chemical bond numbers for PCS/PyC interfaces with different OAs. For the OA = 0° interface, newly formed bonds remain minimal, reflecting the inert nature of the basal plane. In contrast, the evolution of interfacial Cp–C bonds follows a three-stage kinetic profile: (i) Induction period (0–17 ps): the PCS backbone remains largely intact; (ii) Rapid cross-linking phase (17–60 ps): triggered by accelerated Si–C and C–H bond cleavage, flooding the interface with reactive fragments; and (iii) Saturation regime (>60 ps): depletion of edge sites and steric crowding limit further bond formation.

This behavior stems from the coupling of bulk pyrolysis kinetics and interfacial radical transport. While the OA = 85° interface initially forms more Cp–C bonds, its near-perpendicular spatial structure eventually restricts reactions with carbon clusters. Consequently, the OA = 55° interface achieves the highest final bond count, forming a robust C–C molecular skeleton. Across all models, Cp–Si bonds are unstable, acting as transition states that eventually yield to stable C–C bonds. Additionally, H atoms limit the maximum density of interfacial bonding by pre-adsorbing on the interface. Thus, interfacial bond formation is governed by a competitive mechanism between radical accessibility and steric hindrance.

### 3.3. Analysis of a-SiC/PyC Interface Models

**Figure 10:**
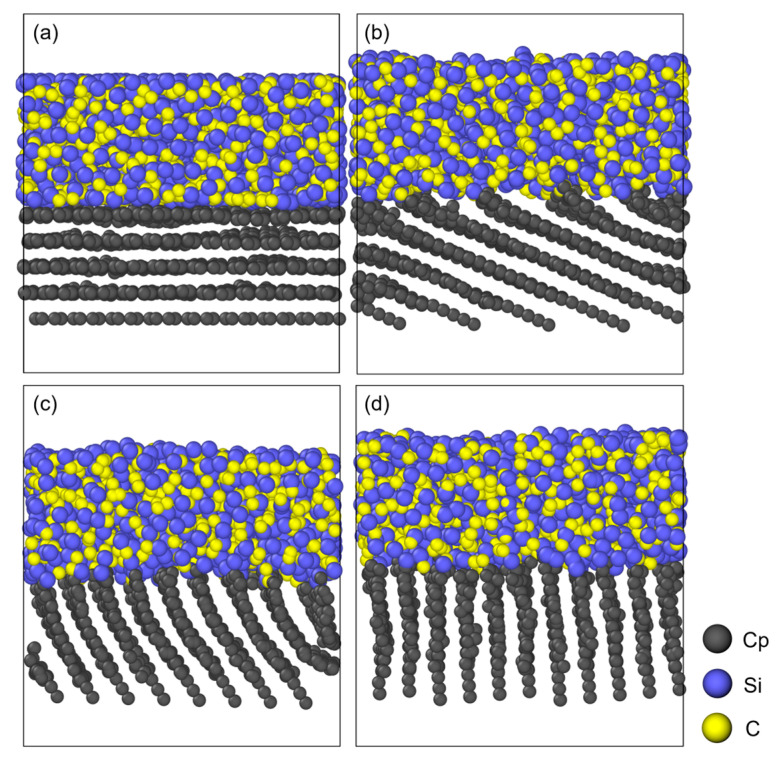
Structural snapshots of amorphous SiC/PyC (a-SiC/PyC) interfaces with different OAs after entire pyrolysis simulation: (**a**) 0°; (**b**) 25°; (**c**) 55°; (**d**) 85°.

**Figure 11:**
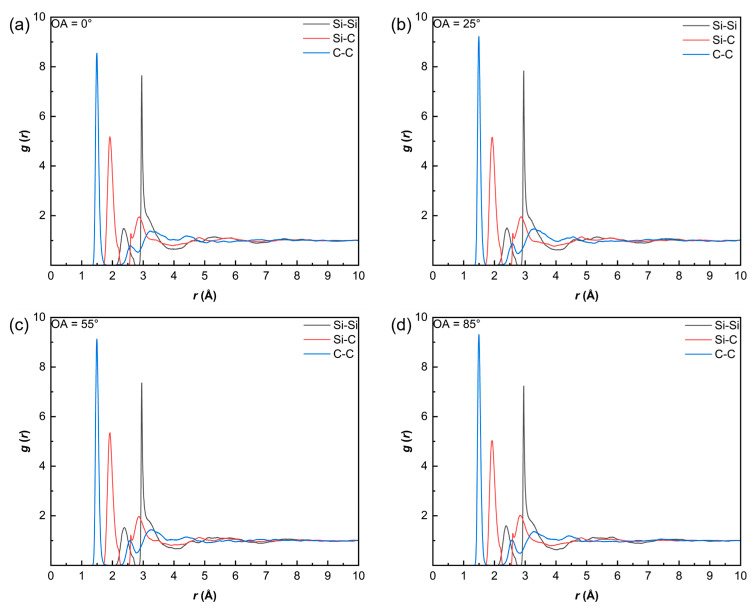
Radial distribution functions (RDF), *g*(*r*), of the a-SiC regions in the four SiC/PyC interface models: (**a**) OA = 0°; (**b**) OA = 25°; (**c**) OA = 55°; and (**d**) OA = 85°.

Through cyclic simulations of small molecule removal and isothermal relaxation, followed by a slow cooling phase, four amorphous SiC/PyC (a-SiC/PyC) interface models are successfully constructed, as shown in Figure 10. And Figure 11 shows the radial distribution functions (RDF) of the a-SiC regions for the four different orientation angles after pyrolysis. These four curves exhibit high consistency. This indicates that although the substrate angle affects the interfacial reactions, the internal microstructure of the a-SiC is mainly determined by the pyrolysis kinetics of the PCS itself.

The products are characterized by amorphous networks with short-range disorder. Given the high carbon content of the PCS precursor C/Si ratio of 1.8–2.0), the products exhibit carbon-rich features, evidenced by significant C–C correlations. This aligns with the “nanodomain model” for polymer-derived ceramics (PDCs), where excess free carbon segregates into a percolating network encapsulating SiC-rich nanodomains. Our MD results capture the early stages of this phase separation. Specifically, the first coordination peak of *g*_Si–C_(*r*) appears at 1.9–2.0 Å, forming the main network skeleton. The *g*_C–C_(*r*) peak at 1.45–1.55 Å suggests sp^3^ hybridized components mixed with sp^2^ hybridization. The *g*_Si–Si_(*r*) peak at 2.9–3.1 Å originates from next-nearest-neighbor correlations within the Si–C–Si structure, with only a weak shoulder at 2.35 Å, confirming that Si–Si bonds are not dominant. These structural features and the calculated RDF peaks of the a-SiC phase are in good agreement with the ReaxFF simulations of hydridopolycarbosilane (HPCS) pyrolysis reported by Naserifar et al. [24]. Furthermore, the dominant Si–C nearest-neighbor distance of 1.9–2.0 Å obtained in our simulations is consistent with the experimental value of 1.89 Å determined by TEM and electron diffraction techniques on amorphous SiC thin films [39]. This agreement with both prior simulations and independent experimental data supports the structural reliability of our thermochemical models.

### 3.4. Analysis of Mechanical Properties of a-SiC/PyC Interfaces

**Figure 12:**
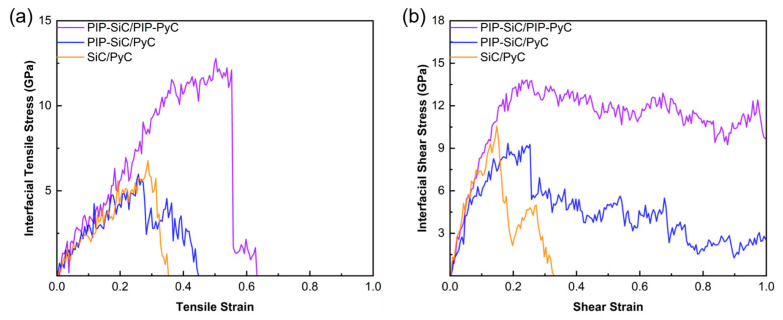
Stress-strain curves of the three different interface models (PIP–SiC/PIP–PyC, PIP–SiC/PyC, and SiC/PyC) during deformation: (**a**) tensile stress–strain curves; (**b**) shear stress-strain curves.

**Figure 13:**
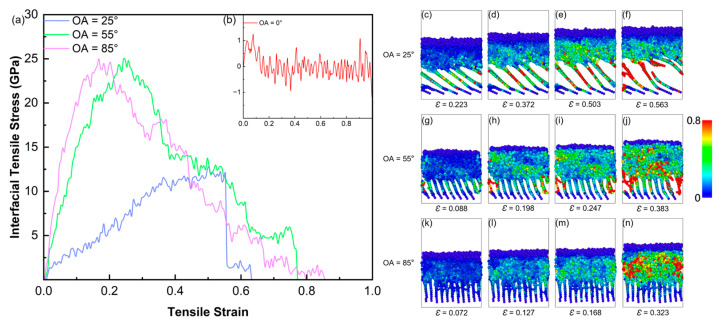
(**a**,**b**) Tensile stress–strain curves of a-SiC/PyC interface models with different OAs; (**c**–**n**) snapshots of the tensile process for three a-SiC/PyC interface models (OA = 25°, 55°, and 85°). Atoms are colored according to von Mises atomic strain.

To evaluate the impact of pyrolysis on mechanical performance, three models were compared: bilateral pyrolysis (PIP–SiC/PIP–PyC), unilateral pyrolysis (PIP–SiC/PyC), and unpyrolyzed (SiC/PyC). As shown in Figure 12, the unpyrolyzed and unilateral models exhibit lower strengths (peak stresses of 6.77 GPa and 5.98 GPa, respectively) and fracture at strains of 0.35–0.45. Notably, the unilateral model (PIP–SiC/PyC) shows a slightly lower peak stress than the unpyrolyzed model (SiC/PyC). This counterintuitive result can be attributed to the structural asymmetry introduced by one-sided pyrolysis: the amorphous SiC produced on a pristine PyC basal surface forms a structurally mismatched interface, and the volumetric shrinkage during ceramization generates residual tensile stress concentrated at this boundary, which offsets the modest gain from the limited number of cross-interfacial bonds formed on the unreconstructed basal plane. In contrast, the bilateral PIP–SiC/PIP–PyC model reaches a peak stress of 12.78 GPa with a fracture strain of 0.55, demonstrating that bilateral pyrolysis promotes covalent bonding and enhances tensile strength. Shear curves (Figure 13b) show a similar trend, where the bilateral model achieves the highest shear strength (13.81 GPa) and maintains high post-fracture stress, indicating superior energy dissipation.

The peak interfacial stresses reported here (up to ~25 GPa in tension) are significantly higher than experimental values for C_f_/SiC composites (≈20–200 MPa). This discrepancy is attributed to: (i) Idealized Models: MD represents atomically flat, defect-free interfaces; (ii) Strain Rates: High rates (10^8^–10^9^ s^−1^) suppress thermally activated defect nucleation, elevating apparent strength to the athermal limit; and (iii) Scale Effects: Small simulation cells preclude long-range stress concentrators. Thus, these values represent theoretical upper bounds of the intrinsic cohesive strength.

Figure 13a,b show the tensile stress–strain curves for the interface models with four different orientation angles (OA = 0°, 25°, 55°, and 85°). For the OA = 0° interface, the tensile stress remains close to 0 GPa throughout the entire loading process (Figure 13b). This result is consistent with the pyrolysis analysis (Section 3.2), which showed that the chemically inert basal plane of the 0° substrate forms negligible cross-interfacial covalent bonds. In the absence of such chemical bridges, the load transfer relies solely on the weak non-bonded interactions between the sp^2^ basal surface and the a-SiC overlayer. Under the Tersoff/AIREBO potential framework employed here, these non-bonded interactions are described implicitly through the short-range repulsive terms and do not capture the full magnitude of dispersive van der Waals forces. Consequently, the near-zero stress for OA = 0° likely represents a lower bound; in reality, a finite but low interfacial strength would be expected even for perfectly basal-oriented PyC due to van der Waals adhesion. This limiting case, however, underscores the fundamental conclusion that covalent cross-linking—rather than physical adhesion—is the primary determinant of interfacial mechanical performance in PIP-processed C_f_/SiC composites.

In contrast, the peak tensile stress of the OA = 25° interface (blue curve) reaches 12.9 GPa. According to the atomic strain snapshots (Figure 13c–f), atoms exhibiting high von Mises local shear strain (visualized in red) are primarily concentrated between the tilted carbon layers. This shows that although the load is transferred from SiC to the PyC substrate, it primarily results in interlaminar peeling and sliding of the PyC layers. Cracks eventually initiate inside the carbon layers.

The OA = 55° (green curve) and OA = 85° (pink curve) interfaces exhibit exceptional tensile resilience, both achieving peak stresses approaching 25 GPa. Crucially, this mechanical enhancement is fundamentally dictated by the preceding pyrolysis dynamics: the high-angle orientations supply a rich density of reactive edge sites, enabling pyrolysis-derived radical fragments to deeply anchor and chemically “suture” the carbon interlayers. Consequently, the failure mechanism transitions from pristine van der Waals sliding to a ductile dissipation process governed by covalent bond scissions. Despite their comparable strengths, their specific failure modes diverge significantly. For the OA = 55° interface (Figure 13g–j), as stretching progresses, the high-strain zones (yellow and red atoms) do not concentrate on a single line but tend to diffuse outwards. As small cracks grow along the interface, their path changes. This spreads the stress over a larger area and this mechanism absorbs fracture energy and improves the toughness of the interface. In contrast, the OA = 85° interface has nearly 90° carbon layers, showing the highest elastic modulus initially. However, the stress becomes highly concentrated in the later loading stage. As clearly seen in Figure 13n, when the external force exceeds the limit, a large number of red atoms (indicating high strain) suddenly appear in the upper SiC region. This indicates that the crack propagates directly across the interface into the bulk SiC matrix, which is characteristic of typical brittle fracture. This difference in failure modes between the 55° and 85° interfaces is caused by their distinct interfacial structures. As discussed in Section 3.2, the strong steric hindrance in the 85° model restricts the deep penetration of the cross-linking networks, which causes highly localized stress concentrations rather than the uniform plastic deformation observed in the 55° model.

**Figure 14:**
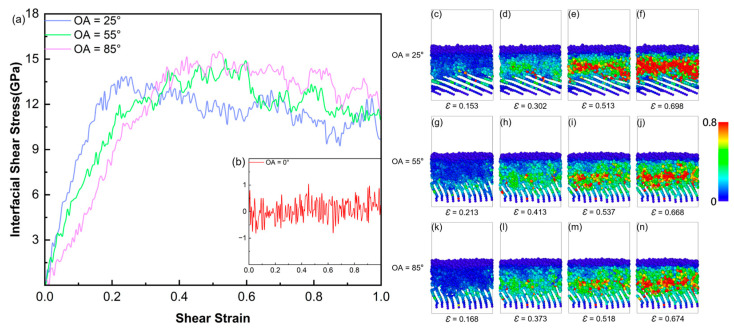
(**a**,**b**) Shear stress–strain curves of a-SiC/PyC interface models with different OAs; (**c**–**n**) snapshots of the shear process for three a-SiC/PyC interface models (OA = 25°, 55°, and 85°). Atoms are colored according to von Mises atomic strain.

Figure 14a,b illustrates the shear stress–strain curves of the interface models with varying orientation angles. For the OA = 0° model, the shear stress remains near 0 GPa (Figure 14b). Consistent with the preceding structural analysis, the parallel carbon layers lack reactive sites; thus, the interface relies primarily on van der Waals forces and is highly susceptible to shear sliding. When the OA is 25°, the peak shear stress of the interface reaches 13.82 GPa. The atomic strain snapshots (Figure 14c–f) reveal that high-strain atoms are concentrated in a localized area near the interface. This indicates improved shear resistance, although the failure mode remains dominated by relative interfacial sliding.

The peak shear stresses of the OA = 55° and 85° models are 15 GPa and 15.49 GPa. Atomic strain snapshots (Figure 14g–n) reveals that they have similar failure mechanisms. During the shear deformation process, the high-strain regions are not confined to a single interfacial slip plane, but extend into the SiC and PyC substrates. This mechanical result is consistent with the previous interfacial reaction mechanism. The higher orientation angles (55° and 85°) provide more PyC edge defect sites, which promotes the formation of cross-interface covalent bonds (such as the sp^3^ cross-linked network). Under shear loading, these chemical bonds strengthen the interface and allow the shear stress to transfer effectively into the substrates. This structural deformation consumes more fracture energy, thereby improving the shear strength of the interface. The simulated transition of failure mode from sliding to plastic fracture agrees well with the MD results of Wang et al. [32]. Furthermore, our models reveal that the pyrolysis-induced cross-linked network is the chemical origin of this transition. This finding provides a theoretical explanation for the interfacial interlocking observed in recent experiments [40].

### 3.5. Effects of Pyrolysis Temperature and Strain Rate on the Mechanical Properties of the OA = 25° Interface

To investigate the effect of pyrolysis temperature and strain rate, the OA = 25° interface was selected as a representative case. This choice was motivated by two considerations: (i) the 25° interface exhibits an intermediate bonding density, making it sensitive to both temperature-driven and rate-driven variations; and (ii) its failure mode—dominated by interlaminar sliding rather than catastrophic brittle fracture—provides a well-defined baseline against which parametric effects can be clearly resolved.

**Figure 15:**
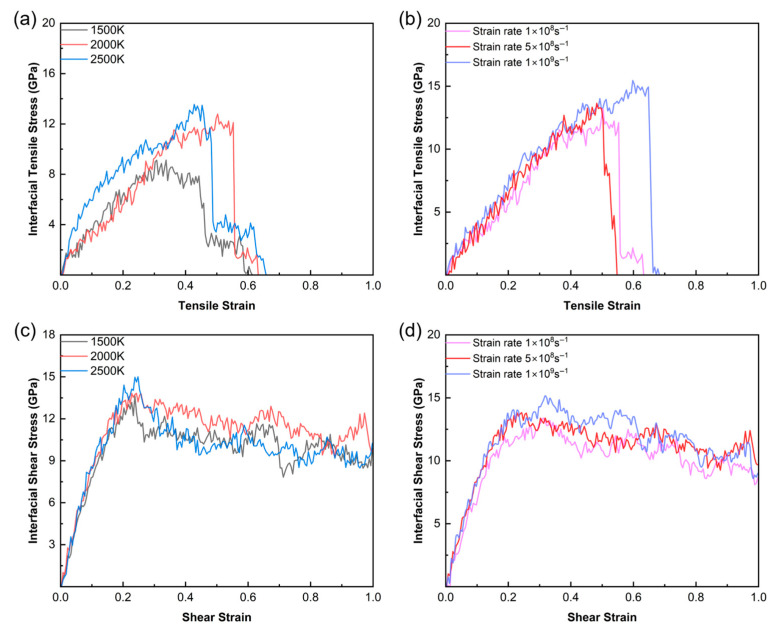
Stress–strain curves of the OA = 25° interface model: tensile curves at different (**a**) pyrolysis temperatures and (**b**) strain rates; shear curves at different (**c**) pyrolysis temperatures and (**d**) strain rates.

Tensile and shear simulations were performed on the OA = 25° interface models generated at three pyrolysis temperatures (1500 K, 2000 K, and 2500 K) at a fixed strain rate of 5 × 10^8^ s^−1^. The stress–strain curves in Figure 15a,c show that both the peak tensile and shear stresses increase with rising pyrolysis temperature. This phenomenon is closely related to the chemical evolution at the interface. At 1500 K, the formation of cross-interface covalent bonds is limited, resulting in relatively weak interfacial bonding. As the temperature increases, more interfacial atoms overcome the reaction potential barriers. This leads to the generation of a higher-density covalent bonding network, thereby improving the load-bearing capacity of the interface.

Furthermore, Figure 15b,d evaluates the effect of different strain rates (1 × 10^8^ s^−1^, 5 × 10^8^ s^−1^, and 1 × 10^9^ s^−1^) on the mechanical response. According to the tensile curves (Figure 15b), as the strain rate increases, the interfacial tensile strength rises from 12.78 GPa to 15.45 GPa. The shear results show a similar trend. This can be attributed to the strain rate effect. At lower strain rates, sufficient time is provided for the structural relaxation of the interface. The interfacial atoms can effectively release stress through interlaminar sliding within the substrate. At higher strain rates, however, the lack of time for structural relaxation results in a higher apparent strength.

## 4. Conclusions

In this study, a coupled thermochemical–mechanical simulation workflow based on ReaxFF molecular dynamics was developed to investigate, for the first time, the dynamic interfacial evolution during the PIP ceramization of PCS precursors on PyC substrates and its direct consequences for interfacial mechanical performance. The principal findings are as follows:

(1) During pyrolysis at 2000 K, the PCS backbone undergoes sequential C–Si bond scission, releasing reactive radical fragments that recombine into an amorphous SiC network interspersed with free carbon clusters. The simulated radial distribution functions of the resulting a-SiC phase—featuring dominant Si–C correlations at 1.9–2.0 Å and C–C correlations at 1.45–1.55 Å—are consistent with the widely accepted nanodomain model for polymer-derived ceramics.

(2) The PyC orientation angle governs the interfacial covalent bridge density through a non-monotonic competition between edge site reactivity and interlayer steric accessibility. The OA = 0° basal surface remains chemically inert, while the OA = 55° configuration maximizes the cross-interfacial Cp–C bond count (~70 bonds after 100 ps) by providing both a high edge site density and sufficient interlayer openness for radical infiltration. The OA = 85° interface, despite exposing the most edge sites, suffers from steric confinement that limits macromolecular penetration.

(3) These pyrolysis-induced bonding topologies dictate the subsequent mechanical failure modes. Under tensile loading, the OA = 55° interface exhibits ductile failure with distributed plasticity and crack deflection (peak stress ~25 GPa), whereas the OA = 85° interface, despite comparable peak strength, fails via brittle crack penetration into the SiC matrix. Under shear loading, both high-OA interfaces (55° and 85°) achieve peak shear stresses of approximately 15 GPa (15.0 and 15.49 GPa, respectively), with high-strain regions extending into the substrates rather than being confined to a single slip plane—a direct consequence of the sp^3^ cross-linked network formed during pyrolysis.

Several limitations should be noted. The accelerated heating rate (10^14^ K/s), nanometer-scale model dimensions (~3.5 nm lateral), and the force field transition from ReaxFF to Tersoff/AIREBO introduce quantitative uncertainties in the absolute stress values. The reported strengths therefore represent theoretical upper bounds for ideal interfaces. Future work should incorporate larger-scale models, explicit validation against ab initio mechanical benchmarks, and multi-cycle PIP simulations to approach more realistic interfacial architectures. Experimental validation through fiber push-out tests combined with in situ Raman spectroscopy of the PyC hybridization state would provide a critical bridge between these atomistic predictions and macroscopic performance.

## Data Availability

The raw data supporting the conclusions of this article will be made available by the authors on request.
